# Lifestyle Interventions with Mind-Body or Stress-Management Practices for Cancer Survivors: A Rapid Review

**DOI:** 10.3390/ijerph20043355

**Published:** 2023-02-14

**Authors:** Acadia W. Buro, Sylvia L. Crowder, Emily Rozen, Marilyn Stern, Tiffany L. Carson

**Affiliations:** 1 Department of Health Outcomes and Behavior, Moffitt Cancer Center, Tampa, FL 33612, USA; 2 Department of Child and Family Studies, College of Behavioral and Community Sciences, University of South Florida, Tampa, FL 33612, USA

**Keywords:** stress, diet, physical activity, cancer survivors, rapid review

## Abstract

This rapid review examined current evidence on lifestyle interventions with stress-management or mind-body practices that assessed dietary and/or physical activity outcomes among cancer survivors. Searches were conducted in PubMed, Embase, and PsycINFO based on Cochrane Rapid Reviews Methods Group rapid review recommendations using the keywords “diet,” “physical activity,” “mind-body,” “stress,” and “intervention.” Of the 3624 articles identified from the initial search, 100 full-text articles were screened, and 33 articles met the inclusion criteria. Most studies focused on post-treatment cancer survivors and were conducted in-person. Theoretical frameworks were reported for five studies. Only one study was tailored for adolescent and young adult (AYA) cancer survivors, and none included pediatric survivors. Nine studies reported race and/or ethnicity; six reported that ≥90% participants were White. Many reported significant findings for diet and/or physical activity-related outcomes, but few used complete, validated dietary intake methods (e.g., 24-h recall; *n* = 5) or direct measures of physical activity (e.g., accelerometry; *n* = 4). This review indicated recent progress on evaluating lifestyle interventions with stress-management or mind-body practices for cancer survivors. Larger controlled trials investigating innovative, theory-based, personalized interventions that address stress and health behaviors in cancer survivors—particularly racial/ethnic minority and pediatric and AYA populations—are needed.

## 1. Introduction

Cancer incidence rates are growing worldwide [1], and five-year survival rates have dramatically increased since the 1970s for several types of cancer [2]. As the number of cancer survivors with comorbid conditions, including hypertension, diabetes, and morbid obesity, has also risen in recent decades [3], lifestyle interventions are needed to reduce the risk of chronic disease and improve the quality of life among cancer survivors [4]. The American Cancer Society and the World Cancer Research Fund/American Institute for Cancer Research recommend healthy eating and physical activity to improve cancer prognosis and survival [5,6]. Adherence to nutrition and physical activity guidelines has been associated with a 10–61% decrease in overall cancer incidence and mortality, with consistent reductions in breast, endometrial, and colorectal cancer, in particular [7].

However, cancer survivors face numerous stressors that may impact their ability to make healthy lifestyle behavior choices. Cancer survivors often experience psychological distress in the form of anxiety, post-traumatic stress disorder (PTSD), fear of cancer recurrence, or depressive symptoms [8]. Psychosocial stress is known to alter dietary and physical activity behaviors in the general population [9,10,11], and prolonged stress may exacerbate endocrine dysfunction, including hypothalamic-pituitary-adrenal (HPA) axis disruption, experienced by many cancer survivors [12]. Mind-body therapies, including meditation and yoga, and other stress-management interventions may help to manage stress [13] and address unmet psychosocial needs among cancer survivors [14]. Yoga, meditation, qigong, mindfulness-based stress reduction (MBSR), and massage have been used as complementary medical therapies for cancer patients [15], and mindfulness-based stress reduction training interventions have been shown to improve the quality of life of cancer survivors [16].

Interventions that address nutrition or physical activity in addition to mind-body therapies may improve healthy lifestyle behaviors among cancer survivors [17,18,19], yet research on such interventions is limited. Given the potential for mind-body therapy and stress reduction to further improve the health and well-being of cancer survivors, there is a need to assess the effects of lifestyle interventions that address such practices in this population. There is a lack of reviews investigating the prevalence of such interventions.

Whereas systematic reviews often take up to two years to complete, rapid reviews streamline systematic review methods to more quickly complete reviews with comparable aims [20]. The purpose of this study was to conduct a rapid review of the literature on the prevalence of lifestyle interventions (i.e., diet and/or physical activity interventions) that address stress-management or mind-body practices and assess diet and/or physical activity-related outcomes among cancer survivors.

## 2. Materials and Methods

### 2.1. Selection Criteria

Searches were conducted in PubMed, Embase, and PsycINFO based on recommendations from the Cochrane Rapid Reviews Methods Group (CRRMG) [20]. The searches were not restricted by publication date. The National Center for Complementary and Integrative Health (NCCIH) mind and body practices (i.e., acupuncture, massage therapy, meditation, relaxation techniques, spinal manipulation, tai chi, and yoga) were used to inform our inclusion criteria for mind-body practices [21]. The inclusion criteria were as follows: (1) participants diagnosed with cancer prior to study enrollment; (2) lifestyle intervention (i.e., diet and/or physical activity intervention) addressing stress-management or mind-body practices; (3) intervention study design (e.g., clinical trial, one-group pre-post); and (4) quantitative measure of dietary intake, physical activity, or physical fitness (e.g., muscular strength, walking distance), including both objective and subjective measures. For our selection criteria, yoga, tai chi, and qigong were defined as mind-body practices rather than physical activity. Studies were limited to publications in English based on CRRMG guidelines [20]. The exclusion criteria were as follows: (1) cancer diagnosis not mentioned in the article’s study inclusion criteria; (2) feeding studies; (3) non-intervention study (e.g., observational, case-control); and (4) protocol paper, conference paper, abstract, letter, or commentary. Multiple papers on the same intervention were allowed if different samples were used in order to describe all samples and outcomes used for each intervention identified. The protocol was registered in PROSPERO during the initial search process (CRD42022307760).

### 2.2. Search Strategy

The search strategy was developed by A.W.B., with initial assistance from a research librarian. The keywords “diet,” “physical activity,” “mind-body,” “stress,” and “intervention” were searched using natural language and controlled vocabulary in PubMed, Embase, and PsycINFO. The Systematic Review Accelerator (SR-Accelerator) from the Institute for Evidence-Based Healthcare at Bond University was used to remove unnecessary terms from the search [22]. For example, NCCIH terms (e.g., “acupuncture,” “massage”) were initially used as keywords, but were removed from the search using the SR-Accelerator. Such terms were still used to identify relevant articles during the screening process. The full search strategy is depicted in Table S1. Screening was conducted according to CRRMG guidelines [20]. The searches were conducted on 16 March 2022.

### 2.3. Screening Process

**Figure 1:**
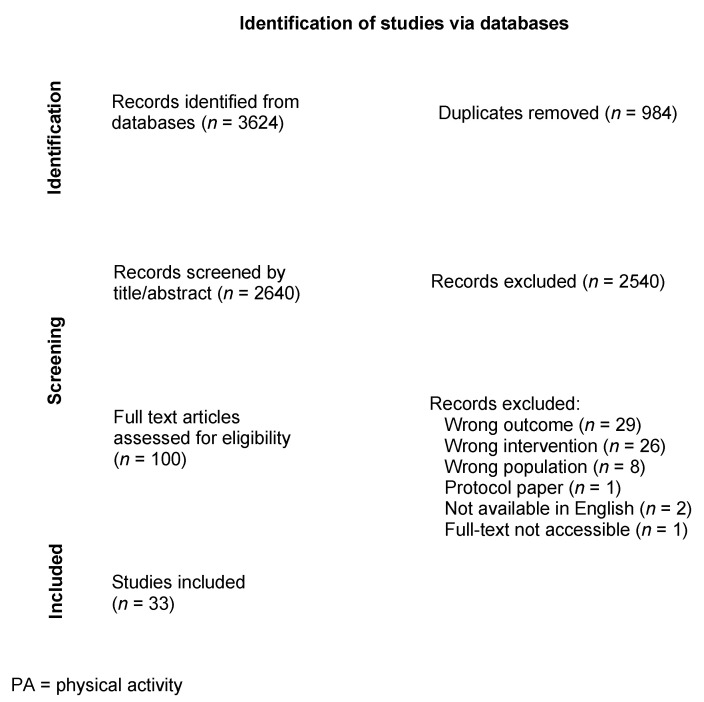
PRISMA 2020 flow diagram for the rapid review process.

After pilot title/abstract screening, all retrieved articles were manually reviewed by A.W.B. based on inclusion and exclusion criteria with a dual screen of 20% of abstracts by S.L.C. After pilot full-text screening, one reviewer (A.W.B.) first screened all full-text articles, and a second reviewer (E.R.) further screened all full-text articles to confirm whether they met the inclusion criteria. Discrepancies were resolved through discussion with a third reviewer (T.L.C.) until a consensus was reached. The PRISMA flowchart for the study is depicted in Figure 1.

### 2.4. Data Extraction

The following data were extracted to Microsoft Excel by a single reviewer (A.W.B.): primary investigator(s), country, publication year, purpose/aim, study design and methods, participant age, type of cancer, timing of study relative to cancer diagnosis, total sample size, intervention and setting, theoretical framework, dietary assessment, physical activity assessment, assessment of psychological (e.g., stress, anxiety, depression) and quality of life (QOL) variables, and study outcomes. A second reviewer (E.R.) checked the extracted data for correctness and completeness. The two reviewers discussed and checked the data extraction together, and a third reviewer (S.L.C.) further reviewed the data extraction results for clarity and completeness.

### 2.5. Study Quality Assessment

Study quality assessment was examined using the National Heart, Lung, and Blood Institute (NHLBI) Study Quality Assessment Tools for controlled intervention studies (14 items) and pre-post studies with no control group (12 items) [23]. Due to the large number of studies identified in this review, two reviewers independently rated the risk of bias (A.W.B. and E.R.) and resolved discrepancies between judgments and support statements through discussion. Quality was categorized as “Good,” “Fair,” or “Poor” based on the guidelines. Mean scores between the two reviewers were considered as a summary score, but the categorization of “Good,” “Fair,” or “Poor” involved consideration of the risk of potential biases beyond the score itself [23]. Studies were not excluded based on quality assessment.

### 2.6. Data Synthesis

Data were synthesized narratively by two reviewers (A.W.B. and E.R.) to demonstrate the overall level of evidence and degree of consistency in findings, with verification of judgments by all other co-authors (S.L.C., M.S., and T.L.C.). Tables were used to summarize the study characteristics. A meta-analysis was not conducted due to the heterogeneity of the studies identified in this review.

## 3. Results

There were 3624 articles identified from the initial search (Figure 1). After screening by title, 984 duplicates were removed, and 2540 were further excluded by title and abstract screening. The remaining 100 articles underwent full-text screening. After further excluding 67 articles, 33 met all criteria for inclusion in the final list.

### 3.1. Study Characteristics

Of the 33 studies included, 16 were pre-post interventions, 13 were randomized controlled trials (RCTs), 3 were pooled analyses of multiple RCTs, and 1 was a quasi-experimental study. The majority of studies were conducted in the US [18,24,25,26,27,28,29,30,31,32,33] or Denmark [19,34,35,36,37,38], with the remaining studies conducted in Canada [39,40,41,42,43], China [44,45,46], South Korea [47,48], Turkey [49], Germany [50], France [51], the Netherlands [52], Australia [53], and Spain [54]. Most studies (58%) were conducted in 2016 or later; all studies were conducted between 2001 and 2022. The participants’ ages ranged from 18 to 85 years, and sample sizes ranged from 9 to 269. Only one study was tailored for adolescent and young adult (AYA) cancer survivors, and none included pediatric cancer survivors. Only nine studies reported race and/or ethnicity, and six of those nine reported that ≥90% participants were White. The studies recruited samples of participants with a range of cancer types (i.e., breast, prostate, lung, ovarian, endometrial, colorectal, melanoma, esophageal, head and neck, and hematological). Eleven studies were not limited to a specific cancer type. Six studies focused on breast cancer survivors. Nineteen studies recruited participants who had completed cancer treatment, nine studies recruited participants before their treatment began, and five studies recruited participants who were undergoing treatment. The characteristics of the studies included are summarized in Table 1.

### 3.2. Overview of Interventions

As some studies discussed the same interventions, there were 26 distinct interventions in this review. Most interventions (*n* = 23) were conducted in-person or did not specify the setting. Some mentioned specific materials, such as a video, booklet, and audio recording [30]. Three included telephone counseling [18,29,48]. Three interventions included diet and mind-body practices, 11 included physical activity and mind-body practices, and 12 included diet, physical activity, and mind-body practices. A few interventions included comprehensive sessions that focused on multiple health behaviors together (e.g., diet, physical activity, and smoking or diet, physical activity, and stress management), rather than including separate components. Diet components included counseling and cooking classes. Mind-body components included complementary therapies (e.g., meditation, yoga, massage, relaxation, Tai Chi) and stress-management counseling. Physical activity components included aerobic and resistance training, walking, and stretching/mobilization. The intervention length ranged from one visit with take-home materials to one year (mean 11 weeks). Theoretical frameworks, reported in five studies, included the Transtheoretical Model [18,26,48], Lazarus and Folkman’s Transactional Model of Stress and Coping [29,40], Social Cognitive Theory [18,26], and Salmon’s unifying theory of physical activity [40]. Descriptions of the interventions are included in Table 2.

### 3.3. Outcomes of Interest

Dietary assessment tools included 24-h (*n* = 3) and 7-day recall (*n* = 1), food frequency questionnaires (FFQs) (*n* = 1), Food Habits Questionnaire (*n* = 2), Mediterranean diet adherence questionnaire (*n* = 1), Block Dietary Fat Screener (*n* = 1), and Block Dietary Fruit, Vegetable, Fiber, and Screener (n = 1). Two studies used questions from validated measures (Block FFQ and “Rules for National Cancer Prevention: Dietary Practice Guideline”).

Fifteen studies used direct physical activity measures only, seven used self-report measures only, and seven used both direct and self-report measures. Physical activity assessment tools included accelerometry (*n* = 4), 7-day Physical Activity Recall (PAR) (*n* = 3), the Community Health Activities Model Program for Seniors (CHAMPS) Physical Activity Questionnaire (*n* = 3), the Godin-Shephard Leisure-Time Physical Activity Questionnaire (GSLTPAQ) (*n* = 1), and step tracking using electronic pedometers (*n* = 1). Physical capacity was evaluated through walking tests (*n* = 14) (6-min (*n* = 11), 12-min (*n* = 1), 50-foot (*n* = 1), and one-mile (*n* = 1) walking tests) and VO_2_ max (*n* = 7) and submax (*n* = 1) tests. Tests for physical strength included hand-held dynamometers measuring grip strength (*n* = 3), the Short Physical Performance Battery (SPPB) (*n* = 3), and one-rep maximums (1RM) (*n* = 2). Tests for flexibility and stability were used in only one study and included the toe touch test, back scratch test, chair-stand test, stand and reach test, single limb stance test, and range of motion (ROM) test. Other measures are included in Table 3.

Psychological and QOL variables were assessed using a variety of surveys/questionnaires. Some questionnaires targeted depression and anxiety, stress, and mindfulness, while others addressed QOL more generally. The most common questionnaires regarding depression and anxiety were the Hospital Anxiety and Depression Scale (HADS) (*n* = 7), Profile of Mood States (POMS) (*n* = 4), and the 10-item Center for Epidemiological Studies-Depression survey (CES-D) (*n* = 2). Stress-specific surveys included both the 4-item (*n* = 1) and 10-item (*n* = 1) Perceived Stress Scale (PSS) and the Stress Reduction Checklist (SRC) (*n* = 1). The most common QOL assessment methods included the 36-Item Short-Form Health Survey (SF-36) (*n* = 8), European Organization for Research and Treatment of Cancer Quality of Life Questionnaire Core 30 (EORTC QLQ-C30) (*n* = 8), Functional Assessment of Cancer Therapy (FACT) General (*n* = 2), Lung (*n* = 2), Multidimensional Fatigue Inventory (MFI-20) (*n* = 2), and Impact of Events Scale (IES) (*n* = 2). Other tools are included in Table 3.

### 3.4. Significant Findings

Of three studies addressing diet and mind-body practices, one reported significant improvements in the animal:vegetable protein ratio (*p* = 0.01) [27] and fruit and vegetable intake (d = 0.23) [31]. One study noted statistically significant improvements in moderate to vigorous physical activity (d = 0.45) and mindfulness (*p* = 0.0039) [31]. None of the studies reported weight-related outcomes. Of 16 studies addressing physical activity and mind-body practices, 7 reported improvements in muscular strength (*p* < 0.05) [19,35,37,38,47,49,51], 6 reported improved walking distance on the 6-min walk test (6MWT; *n* = 5) [38,45,46,49,50,51] or the 12-min walk test (*n* = 1) [47] (*p* < 0.05), 5 reported increased PA levels (*p* ≤ 0.02) [18,26,30,35,36], and 5 reported improved depression scores (*p* ≤ 0.04) [30,34,36,45,46]. Thirteen reported significant improvements in psychological and/or quality of life outcomes (Table 3). Only one measured a weight-related outcome and reported a significant decrease in skin-fold measurements by 3% (*p* = 0.031) [37]. Of 14 studies that addressed diet, physical activity, and mind-body practices, 5 studies reported improvements in healthy eating (e.g., fruit and vegetable intake, fat intake; *p* ≤ 0.03) [25,28,29,32,54], and 3 reported improved 6MWT performance (*p* ≤ 0.01) [39,42,43]. Significant improvements for psychological and/or quality-of-life outcomes were reported in eight studies (Table 3). Nine assessed weight-related outcomes; five reported significant findings regarding BMI, weight, and/or lean body mass [32,33,43,44,54]. Other relevant significant findings are summarized in Table 3.

### 3.5. Quality Assessment

The 16 controlled intervention studies had a mean quality rating of 10/14, with eight studies rated as Good and eight rated as Fair. Six had ≥20% attrition or did not report a dropout rate, and seven had inadequate details on their randomization method. Among the 17 pre-post-intervention studies, 10 were rated as Good, and seven were rated as Fair; only one had sufficient sample size to provide confidence in findings; only one mentioned that those assessing outcomes were blinded to the exposure/intervention; and only two used an interrupted time-series design. The mean rating was 7.8/12. The results of the quality assessment are summarized in Table 4.

## 4. Discussion

This review identified 33 studies on lifestyle interventions (diet and/or physical activity) that addressed stress-management or mind-body practices for cancer survivors. Most studies focused on post-treatment cancer survivors, were conducted in-person, and/or were conducted with participants from a wide age range. None included pediatric cancer survivors, and only one intervention was tailored for AYA survivors. Nine studies reported participants’ race and/or ethnicity, and all nine indicated that ≥70% of the participants were White. Many reported significant findings for diet and/or physical activity-related outcomes, but few used complete, validated dietary intake methods (e.g., 24-h recall; *n* = 5) or direct measures of physical activity (e.g., accelerometry; *n* = 4). All studies identified in this review were categorized as Good or Fair quality based on NHLBI Study Quality Assessment Tools. However, most pre-post interventions were limited by a small sample size, and 63% of controlled interventions had high attrition (≥20%) or did not report attrition.

The findings indicated a lack of interventions for pediatric and AYA cancer survivors. Compared to other cancer survivors, AYA cancer survivors have an increased risk of anxiety, cancer-related worry, post-traumatic stress symptoms [55], and mood disturbances [8]. Although lifestyle interventions show promise for changing health behaviors among pediatric and AYA cancer survivors [56,57], existing interventions do not address the burden of psychosocial stress, which may impact these behaviors. Research is warranted to adequately tailor interventions for younger populations that have unique psychosocial and developmental needs.

Most of the studies with one health behavior component (i.e., diet or physical activity) assessed behavioral outcomes and reported significant findings. Even though 14 studies (42%) included comprehensive interventions that incorporated diet, physical activity, and mind-body practices, 10 of the 14 (71%) did not report diet and/or physical activity outcomes. Future research may incorporate multiple health behaviors into their intervention evaluation, as well as intervention design, to adequately assess behavior change. Six of the nine studies that assessed weight-related outcomes reported significant findings, indicating a need for larger studies with metabolic outcomes (e.g., blood glucose levels, blood pressure).

The findings highlight a need for rigorous measures of diet and physical activity for interventions. Real-world dietary intake is often assessed with self-report measures, such as 24-h dietary recalls, food records, and FFQs [58]. Many studies in this review used screeners or selected questions from validated measures; only five used dietary recall or FFQs. None used Weighed Food Records (WFR), the “gold standard” for dietary assessment [59]. More studies (*n* = 22) used direct measures of physical activity; seven studies collected self-reported data as their only source of physical activity data. None of the studies used bio-behavioral assessments for psychological and QOL variables, such as cortisol, to measure stress.

The limitations of this review included the rapid review methodology. Although much of the rapid review methodology is similar to that of a systematic review, relevant studies may have been missed due to the limitations of a rapid review (e.g., not reviewing journal indexes or gray literature; the second reviewer screened only 20% of identified studies, including only three databases). Additionally, studies were restricted to those published in English, limiting the generalizability of findings. A meta-analysis was not conducted due to heterogeneity regarding age, cancer type, time since treatment, outcomes, intervention type, and length. Although this review focused on lifestyle interventions that address stress-management or mind-body practices for cancer survivors, the authors recognize that characterizing yoga, tai chi, and qigong as mind-body practices rather than physical activity may limit finding interpretability across physical activity outcomes. These activities were characterized based on the NCCIH definition of mind and body practices. [21] Future reviews may examine the effects of yoga, tai chi, and qigong interventions alone on physical activity outcomes. Additionally, there was reporting bias in this review, as multiple papers on the same intervention were included if different samples and outcomes were used. These articles were grouped together in Table 2 for transparency.

Despite its limitations, this review followed the CRRMG recommendations for conducting rapid reviews to complete a streamlined synthesis of knowledge on lifestyle interventions that address stress-management or mind-body practices among cancer survivors. To analyze the 33 studies identified, the research team adopted systematic review protocols for study quality assessment and data synthesis and used a longer, more rigorous approach to data extraction than is recommended by the CRRMG.

## 5. Conclusions

This review indicated recent progress on evaluating lifestyle interventions that address stress-management or mind-body practices among cancer survivors. The results suggest a need for larger controlled studies with standardized measures of psychological, behavioral, and metabolic outcomes. The findings further highlight a need for tailored interventions, specifically for racial/ethnic minority survivors and pediatric and AYA survivors, who are underrepresented in the literature. Further research should investigate innovative, theory-based, personalized interventions that address stress and health behaviors among cancer survivors. Future studies may also compare the effects of various lifestyle interventions with and without stress-management or mind-body practices (e.g., whether interventions targeting multiple health behaviors are more effective at improving mental health outcomes than interventions targeting a single health behavior or whether lifestyle interventions with stress-management or mind-body practices are more effective at improving health behaviors than lifestyle interventions alone).

## Figures and Tables

**Table 1:** Features of the studies included.

Study	Study Design	Country	Sample Size	Participant Age (Range or Mean)	Participant Race/Ethnicity	Participant Cancer Type	Participant Time Since Treatment
Saxe, 2001 [25]	PPI	USA	10	61–78 (mean 67)	NR	Prostate	≥6 months post-treatment
Andersen, 2004 [26]	RCT	USA	227	20–85	90% White	Breast	Pre-adjuvant therapy
Midtgaard, 2005 [35]	PPI	Denmark	91	18–65 (mean 43)	NR	Non-specific	Undergoing cytostatic treatment
Adamsen, 2006 [36]	PPI	Denmark	82	18–63	NR	Non-specific	≥1-month post-diagnosis, admitted for outpatient chemotherapy
Midtgaard, 2006 [37]	PPI	Denmark	61	18–65 (mean 43)	NR	Non-specific	Undergoing cytostatic treatment
Quist, 2006 [38]	PPI	Denmark	70	18–65 (mean 43)	NR	Non-specific	≥1-month post-diagnosis, admitted for outpatient chemotherapy
Fillion, 2008 [41]	RCT	Canada	87	Mean 52	NR	Breast	≤2 years post-initial treatment
Adamsen, 2009 [20]	RCT	Denmark	269	20–65 (mean 47)	NR	Non-specific	Undergoing adjuvant chemotherapy or treatment
Rabin, 2009 [27]	PPI	USA	23	21+ (mean 53)	96% non-Hispanic White, 4% Hispanic White	Breast	Post-treatment
Carmody, 2012 [28]	RCT	USA	36	69	91% non-Hispanic White	Prostate	Post-primary treatment
Hébert, 2012 [29]	RCT	USA	47	70–71 ^a^	70% White, 30% Black or African American	Prostate	Post-treatment
Quist, 2012 [39]	PPI	Denmark	29	45–80 (mean 63)	NR	Lung	Undergoing chemotherapy
Garrett, 2013 [30]	PPI	USA	46	22+ (mean 60)	89% White	Non-specific	≥1-year post-treatment
Jacobsen, 2013 [31]	RCT	USA	286	Mean 57–58 ^a^	≥90% White and ≥83% non-Hispanic per group	Non-specific	Scheduled to receive chemotherapy
Vardar Yağlı, 2015 [50]	RCT	Turkey	52 (statistical analyses on 40)	20–60 (mean 47–50 ^a^)	NR	Breast	≥3 years post-treatment
Hwang, 2016 [48]	PPI	South Korea	40	30+	NR	Ovarian	6 months–3 years in remission
Rabin, 2016 [19]	RCT	USA	35	18–39 (mean 34)	74% White	Non-specific	Post-treatment
Lucas, 2017 [32]	PPI	USA	17	45–70 (mean 61)	88% White	Endometrial	Post-treatment
Yun, 2017 [49]	RCT	South Korea	248	20+ (mean 51)	NR	Non-specific	<2 years post-treatment
Beumeler, 2018 [53]	PPI	Netherlands	9	34–74	NR	Non-specific	≥6 months post-diagnosis, stable long-term
Awasthi, 2019 [42]	P	Canada	150	Mean 66–70 ^a^	NR	Colorectal	Scheduled for colorectal resection of localized cancer
Barrett-Bernstein, 2019 [43]	P	Canada	172	18+ (mean 67)	NR	Colorectal	Awaiting resection
Gillis, 2019 [44]	P	Canada	139	18+ (mean 66–69 ^a^)	NR	Colorectal	Awaiting resection
Lacey, 2019 [54]	PPI	Australia	28	42–85 (median 66)	NR	Melanoma	Undergoing standard treatment with pembrolizumab
Schneeberger, 2019 [33]	PPI	USA	21	41–73 (mean 56)	NR	Breast	Post-treatment
Thomas, 2019 [34]	RCT	USA	51	29–76 (mean 58)	96% White	Non-specific	Active or in remission
Cheng, 2020 [45]	PPI	China	20	18–75 (mean 62)	NR	Esophageal	Scheduled for esophageal radical resection
Felser, 2020 [51]	PPI	Germany	12	52–81 (mean 68)	NR	Head and neck	Post-treatment
Lu, 2020 [46]	PPI	China	16	44–63	NR	Lung	6–12 weeks post-treatment
Ruiz-Vozmediano, 2020 [55]	RCT	Spain	63	18+ (mean 48–51^a^)	NR	Breast	>1-year post-treatment
Ma, 2021 [47]	3-arm RCT	China	101	37–72, (mean 55–58^a^)	NR	Lung	Scheduled for thoracoscopic surgery
Minnella, 2021 [40]	RCT	Canada	70	Mean 70 (intervention), 66 (control)	NR	Bladder	Scheduled for elective radical cystectomy
Fournié, 2022 [52]	PPI	France	17	54.5	NR	Hematological malignancies	≤6 months post-treatment remission

NR = not reported; P = pooled analysis of two or more RCTs; PA = physical activity; PPI = pre-/post-intervention; RCT = randomized controlled trial. ^a^ Group means.

**Table 2:** Description of interventions.

Study	Diet	Physical Activity	Mind-Body	Length of Intervention
Diet & Mind-Body				
Saxe, 2001 [25]	Individual dietary counseling, instruction, and practice in preparing dishes	N/A	Mindfulness meditation training, yoga, social support	12 weeks
Carmody, 2012 [28]	Dietary and cooking classes	N/A	Mindfulness training (e.g., sitting meditation, stretching, mindful awareness)	11 weeks
Lucas, 2017 [32]	Dietary counseling	N/A	Mindfulness-based intervention emphasizing mindful yoga	8 weeks (+6 weeks at home)
PA & Mind-Body				
Midtgaard, 2005 [35]; Adamsen, 2006 [36]; Midtgaard, 2006 [37]; Quist, 2006 [38]; Adamsen, 2009 [20]; Quist, 2012 [39]	N/A	Strength training, cardiovascular exercise	Relaxation, body awareness, massage	6 weeks
Fillion, 2008 [41]	N/A	Supervised walking training, personalized walking program	Psycho-educative fatigue management sessions (e.g., relaxation, self-regulation)	4 weeks
Rabin, 2009 [27]	N/A	Walking	Progressive muscle relaxation	12 weeks
Jacobsen, 2013 [31]	N/A	Guidance and materials on paced breathing, progressive muscle relaxation with guided imagery, and coping self-statements to manage stress	Guidance and materials on regular exercise with an emphasis on walking	One-time meeting + materials with follow-up at 6-and 12-weeks post-intervention
Vardar Yağlı, 2015 [50]	N/A	Treadmill aerobic exercise	Yoga (asanas, pranayama, awareness meditation, and relaxation techniques)	6 weeks
Hwang, 2016 [48]	N/A	Exercise instruction	Relaxation therapy instruction	8 weeks
Rabin, 2016 [19]	N/A	Guidance on increasing moderate-intensity aerobic exercise	Meditation CD (sitting meditation, body scan, yoga stretches)	12 weeks
Felser, 2020 [51]	N/A	Warm-up/mobilization, coordination exercises, resistance exercises, stretching	Relaxation exercises (e.g., progressive muscle relaxation)	12 weeks
Lu, 2020 [46]	N/A	Brisk walking and resistance exercises with major limb movements	Tai chi adapted to 8 simple styles for strength, mobility, and range of motion	12 weeks
Ma, 2021 [47]	N/A	Brisk walking on a treadmill before surgery and climbing stairs after surgery	Inspiratory muscle training and incentive deep-breathing exercise	2 weeks
Fournié, 2022 [52]	N/A	Aerobic and resistance training at moderate intensity	Heart rate variability biofeedback	12 weeks
Diet, PA, & Mind-Body				
Andersen, 2004 [26]	Sessions on health behaviors (diet, PA, smoking)	Sessions on health behaviors (diet, PA, smoking)	Sessions on stress and QOL	18 weeks
Hébert, 2012 [29]	Food-related goal setting, cooking	Individualized PA goal setting	MBSR	6 months
Garrett, 2013 [30]	Telephone counseling	Telephone counseling	Telephone counseling on uncertainty and stress management	3 months
Yun, 2017 [49]	Health education workshop (PA, diet, and distress management) and telecoaching	Health education workshop (PA, diet, and distress management) and telecoaching	Health education workshop (PA, diet, and distress management) and telecoaching	6 months (follow-up at 12 months)
Beumeler, 2018 [53]	Dietitian and group sessions	Physical therapy sessions	Mind-body therapy led by physical therapists	12 months
Awasthi, 2019 [42]; Barrett-Bernstein, 2019 [43]; Gillis, 2019 [44]	Nutritional counseling with whey protein supplementation	Aerobic and resistance exercise	Anxiety reduction strategies (e.g., diaphragmatic breathing)	4 weeks before surgery and 8 weeks after
Lacey, 2019 [54]	Tailored dietary changes	Exercise classes and activity monitoring	Complementary therapies (massage, reflexology yoga, qigong, mindfulness meditation, or acupuncture)	8 weeks
Schneeberger, 2019 [33]	One dietitian-led educational visit and two culinary medicine visits in a teaching kitchen	PA instructions (i.e., on aerobic and strength exercises) incorporated into each visit	One visit on stress relief (e.g., guided imagery, mindful sitting and eating) and one visit on yoga poses and breathing	14 weeks
Thomas, 2019 [34]	Nutritional counseling sessions	Group exercise sessions	Training in mindfulness, reappraisal, and savoring	10 weeks
Cheng, 2020 [45]	Nutrition guidance for total and partial enteral nutrition and oral nutrition periods	Inspiratory muscle training, walking	MBCR courses, Baduanjin Qigong (defined as physical activity)	12 weeks
Ruiz-Vozmediano, 2020 [55]	Workshops on healthy eating	Classes with circuits and stretching, as well as dance classes	MBSR sessions focused on meditation and yoga	6 months
Minnella, 2021 [40]	Dietitian-created food-based intervention with personalized energy and macronutrient requirements	Individualized, home-based, moderate-intensity aerobic and resistance training	Psychosocial counseling session with training on relaxation and imagery techniques, including passive breathing exercise, meditation skills, and guided imagery	Not reported

MBCR = mindfulness-based cancer recovery; MBSR = mindfulness-based stress reduction.

**Table 3:** Assessment tools and significant findings for outcomes of interest.

Study	Diet	PA	Psychological or QOL
Diet & Mind-Body			
Saxe, 2001 [25]	7-Day Dietary Recall; no statistical tests conducted	Ancillary questions on the 7-Day Dietary Recall; no statistical tests conducted	None
Carmody, 2012 [28]	24-h recall; decreased animal:vegetable protein ratio (*p* = 0.01)	N/A	Reported elsewhere
Lucas, 2017 [32]	FHQ; intervention completers analysis showed small to moderate effect size for change in fruit and vegetable intake (d = 0.23)	Accelerometer, PAQ, SPPB; intervention completers analysis showed moderate effect for MVPA (d = 0.45)	FFMQ, MAAS, SWLS, SF-36; Intervention completers analysis showed improved mindfulness (*p* = 0.0039)
PA & Mind-Body			
Midtgaard, 2005 [35]	N/A	VO₂ max; improved (*p* < 0.001)	HADS; anxiety (*p* = 0.001) and depression (*p* = 0.042) reduced
Adamsen, 2006 [36]	N/A	1RM and VO₂ max; improved muscular strength (*p* < 0.001), physical fitness (*p* < 0.001) and PA levels (*p* < 0.001)	EORTC QLQ-C30, SF-36; improved physical functioning (*p* < 0.001) and role functioning (*p* < 0.001)
Midtgaard, 2006 [37]	N/A	Interview and quantitative assessment for leisure time PA, developed by Saltin and Grimby (1968); PA significantly improved at 1 and 3 months post-intervention (*p* < 0.0001)	HADS; depression was significantly reduced (*p* = 0.0016)
Quist, 2006 [38]	N/A	1RM and VO₂ max; increased muscular strength, aerobic fitness, and VO₂ max (*p* < 0.001)	None
Fillion, 2008 [41]	N/A	VO₂ submax, accelerometer; no significant differences	POMS, SF-12; improved emotional distress (d = 0.33) and quality of life (d = 0.34)
Adamsen, 2009 [20]		Questionnaire by Aadahl and Jørgensen (2003), 1RM and VO₂ max; maximum oxygen consumption and muscular strength improved (*p* < 0.0001)	EORTC QLQ-C30, SF-36; improved fatigue (d = 0.33), vitality (d = 0.55) physical functioning (d = 0.37), role physical (d = 0.37), role emotional (d = 0.32), and mental health (d = 0.28) scores
Rabin, 2009 [27]	N/A	PAR, accelerometer; increased moderate- intensity activity (*p* < 0.0001) and energy expenditure (*p* < 0.001)	POMS; reduced tension/anxiety and overall mood disturbance (*p* < 0.05)
Quist, 2012 [39]	N/A	VO₂ max, 6MWT, 1RM; improved VO₂ max (*p* = 0.014), 6MWT distance (*p* = 0.006), muscular strength (*p* < 0.05)	FACT-L; emotional well-being improved (*p* = 0.03, d = 0.38)
Jacobsen, 2013 [31]	N/A	GSLTPAQ; improved (*p* = 0.022)	SF-36, CES-D, 21-item BAI, SRC; decreased depression (*p* = 0.019), anxiety (*p* = 0.049), and stress (*p* = 0.013)
Vardar Yağlı, 2015 [50]	N/A	6MWT, hand-held dynamometer; improved 6MWT distance and peripheral muscular strength (*p* < 0.05)	EORTC QOL-C30; QOL improved (*p* < 0.05)
Hwang, 2016 [48]	N/A	12-min walk, chair-stand test; improved walking distance (*p* = 0.005) and muscular strength (*p* = 0.001)	FACT-G; physical, social/familial, emotional, and functional well-being subscales (*p* ≤ 004)
Rabin, 2016 [19]	N/A	PAR, one-mile walk test, accelerometer; moderate-intensity PA (*p* = 0.002) and cardiovascular fitness (*p* = 0.008)	POMS; no significant change in overall mood disturbance
Felser, 2020 [51]	N/A	6MWT, SPPB, ROM, stand and reach; improved head rotation (*p* = 0.042), walking distance (*p* = 0.010), and SPPB score (*p* = 0.031)	EORTC QLQ-30; overall difference not significant; positive changes in physical function (*p* = 0.008), cognitive function (*p* = 0.015), and social function scales (*p* = 0.031)
Lu, 2020 [46]	N/A	6MWT, SPPB, electronic pedometer, FEV; improved FEV (*p* = 0.02)	SAS, SDS, EORTC QLQ-C30; improved emotional functioning (*p* = 0.012), SAS (*p* = 0.002), and SDS (*p* = 0.02) scores
Ma, 2021 [47]	N/A	6MWT; improved exercise capacity (*p* = 0.028)	HADS; depression reduced (*p* = 0.002)
Fournié, 2022 [52]	N/A	6MWT, 50-foot walk test, grip force test, single limb stance test, toe touch and back scratch test; improved 6MWT and 50-foot walk test (*p* < 0.001), muscular strength (*p* ≤ 0.01), flexibility (*p* ≤ 0.01)	MFI-20; no significant differences
Diet, PA, & Mind-Body			
Andersen, 2004 [26]	FHQ; improved dietary patterns (*p* = 0.03)	PAR; no significant differences	POMS, IES; Total Mood Disturbance (*p* = 0.04) and anxious moods (*p* = 0.04) decreased
Hébert, 2012 [29]	24-h recall; decreased saturated fatty acid (*p* < 0.0001), total energy (*p* = 0.01), and total fat (*p* = 0.02) intake	CHAMPS; increased PA at 3 (*p* = 0.009) and 6 months (*p* = 0.08)	None
Garrett, 2013 [30]	Two questions from the Block FFQ; fruit and vegetable consumption increased *(p* = 0.02)	Standardized questions from the BRFSS; PA increased (*p* = 0.006, *p* = 0.01)	IES; cancer-specific distress decreased (*p* < 0.001)
Yun, 2017 [49]	Validated questions based on the “Rules for National Cancer Prevention: Dietary Practice Guideline”; no difference in vegetable intake	Survey responses about the time, length, and intensity of PA; no difference in METS	HADS, EORTC QLQ-C30; anxiety decreased at 3 months (*p* = 0.025) but not 12 months
Beumeler, 2018 [53]	None	Grip strength, bicep curl, steep ramp; bicep curl increased (*p* = 0.043)	DASS-21, SF-36, VAS-F: anxiety (*p* = 0.011), stress (*p* = 0.017), and role limitations due to physical health (*p* = 0.034) and emotional problems (*p* = 0.016) decreased
Awasthi, 2019 [42]	None	6MWT and muscular strength; improved functional capacity and muscular strength (*p* < 0.01)	HADS, SF-36; mental and physical QOL improved (*p* < 0.0001)
Barrett-Bernstein, 2019 [43]	None	6MWT, CHAMPS; improved 6-min walking distance (*p* = 0.006)	SF-36, HADS; no statistical comparisons
Gillis, 2019 [44]	None	6MWT; no statistical comparisons	None
Lacey, 2019 [54]	None	Steps, 1RM row and leg press, exercise hours, VO₂ max; no statistical comparisons	FACT-G, HADS, ESAS; no statistical comparisons
Schneeberger, 2019 [33]	Block Dietary Fat Screener and Block Dietary Fruit, Vegetable, Fiber, and Screener; reduced weekly fat consumption (*p* < 0.01)	None	PSS-4, CES-D, PROMIS; no significant differences
Thomas, 2019 [34]	N/A	None	DEBQ, MAIA; significant time × treatment interaction for eating in response to external cures (*p* = 0.01), improvements in noticing body sensations (*p* = 0.01), attention regulation (*p* = 0.02), self-regulation (*p* = 0.006), and body listening (*p* = 0.001)
Cheng, 2020 [45]	None	6MWT; no significant changes	EORTC QLQ-C30, PHQ-9, GAD-7, and PSS-10; depression increased 1-month after surgery (*p* < 0.001); most QOL dimensions returned to preoperative level at 3-month follow-up
Ruiz-Vozmediano, 2020 [55]	FFQ, 24 h recall, 14-item Mediterranean diet adherence questionnaire; improved Mediterranean diet adherence (*p* = 0.02)	None	EORTC QLQ-C30; improved physical functioning (*p* = 0.027) and role functioning (*p* = 0.028)
Minnella, 2021 [40]	None	6MWT, CHAMPS; 6MWT difference between groups at 4 weeks (*p* = 0.014) but no differences in reported PA	SF-36; no significant differences

1RM = One-repetition maximum; 6MWT = 6-Minute Walk Test; BAI = Beck Anxiety Inventory; BRFSS = Behavioral Risk Factor Surveillance System; CES-D 10 = 10-item Center for Epidemiological Studies-Depression survey; CHAMPS = Community Healthy Activities Model Program for Seniors; DASS-21 = Depression, Anxiety and Stress Scale-21 Items; DEBQ = Dutch Eating Behavior Questionnaire; ESAS = Edmonton Symptom Assessment System; EORTC QLQ-C30 = European Organization for Research and Treatment of Cancer Quality of Life Questionnaire Core 30; FACT-G = Functional Assessment of Cancer Therapy–General; FACT-L = Functional Assessment of Cancer Therapy–Lung; FEV = forced expiratory volume; FFMQ = 5-Facet Mindfulness Questionnaire; FFQ = Food Frequency Questionnaire; FHQ = Food Habits Questionnaire; GAD-7 = General Anxiety Disorder-7; GSLTPAQ = Godin-Shephard Leisure-Time Physical Activity Questionnaire; HADS = Hospital Anxiety and Depression Scale; IES = Impact of Events Scale; MAAS = Mindfulness Attention Awareness Scale; MAIA = Multidimensional Assessment of Interoceptive Awareness; METS = metabolic equivalents of task; MFI-20 = Multidimensional Fatigue Inventory; MVPA = moderate to vigorous physical activity; PA = physical activity; PAR = 7-day Physical Activity Recall; PAQ = Paffenbarger Physical Activity Questionnaire; PHQ-9 = Patient Health Questionnaire-9; POMS = Profile of Mood States; PROMIS = Patient-Reported Outcomes Measurement Information System; PSS-4 = 4-item Perceived Stress Scale; PSS-10 = Perceived Stress Scale 10; QOL = quality of life; ROM = range of motion; SAS = Self-rated Anxiety Scale; SDS = Self-rating Depression Scale; SF-12 = 12-Item Short-Form Health Survey; SF-36 = 36-Item Short-Form Health Survey; SPPB = Short Physical Performance Battery; SRC = Stress Reduction Checklist; SWLS = Satisfaction with Life Scale; VAS-F = visual analog scale-fatigue; VO₂ max = maximal oxygen consumption.

**Table 4:** Quality assessment ratings.

Study	Category	Score
Controlled Interventions		
Andersen, 2004 [26]	G	11.5
Fillion, 2008 [41]	G	12
Adamsen, 2009 [20]	G	12
Carmody, 2012 [28]	F	10
Hébert, 2012 [29]	F	9
Jacobsen, 2013 [31]	F	9
Vardar Yağlı, 2015 [50]	F	8.5
Rabin, 2016 [19]	F	8
Yun, 2017 [49]	F	10
Awasthi, 2019 [42]	F *	5
Barrett-Bernstein, 2019 [43]	F	7
Gillis, 2019 [44]	G	11
Thomas, 2019 [34]	G	11.5
Ruiz-Vozmediano, 2020 [55]	G	12
Ma, 2021 [47]	G	11.5
Minnella, 2021 [40]	G	12.5
Pre-post Interventions		
Saxe, 2001 [25]	F	7
Midtgaard, 2005 [35]	G	8
Adamsen, 2006 [36]	F	7.5
Midtgaard, 2006 [37]	G	9
Quist, 2006 [38]	F	7.5
Rabin, 2009 [27]	G	8
Quist, 2012 [39]	F	7
Garrett, 2013 [30]	F	7
Hwang, 2016	G	10
Lucas, 2017 [32]	F	7
Beumeler, 2018 [53]	G	8
Lacey, 2019 [54]	F	6
Schneeberger, 2019 [33]	G	8.5
Cheng, 2020 [45]	G	8
Felser, 2020 [51]	G	8
Lu, 2020 [46]	G	8
Fournié, 2022 [52]	G	8

G = good; F = fair; * Some information reported in another paper.

## Data Availability

Not applicable.

## Supplementary Materials

The following supporting information can be downloaded at: https://www.mdpi.com/article/10.3390/ijerph20043355/s1, Table S1. Full Search Strategy.
