# Supplementary material for: Lifestyle Interventions with Mind-Body or Stress-Management Practices for Cancer Survivors: A Rapid Review
Source: Int J Environ Res Public Health. 2023 Feb 14;20(4):3355. doi: 10.3390/ijerph20043355 (PMC9964062; doi:10.3390/ijerph20043355)
Supplement: Supplementary file 1 [file ijerph-20-03355-s001.zip › ijerph-2172651-supplementary.pdf]

Table S1. Full Search Strategy

PubMed
(diet[Title/Abstract] OR dietary[Title/Abstract] OR diets[Title/Abstract] OR nutrition[Title/Abstract] OR eating[Title/Abstract] OR Diet[MeSH Terms] OR “Nutritional Status”[MeSH Terms] OR “Nutrition Therapy”[MeSH Terms] OR diet[Other Term] OR dietary[Other Term] OR diets[Other Term] OR nutrition[Other Term] OR eating[Other Term] OR “physical activity”[Title/Abstract] OR exercis*[Title/Abstract] OR “physical fitness”[Other Term] OR Exercise[MeSH Terms] OR “physical activity”[Other Term] OR “physical fitness”[Other Term] OR exercis*[Other Term]) AND (“mind-body”[Title/Abstract] OR “mind body”[Title/Abstract] OR mindful*[Title/Abstract] OR relax*[Title/Abstract] OR meditat*[Title/Abstract] OR stress*[Title/Abstract] OR “Mind-Body Therapies”[MeSH Terms] OR “Stress, Psychological”[MeSH Terms] OR “mind-body”[Other Term] OR mindfulness[Other Term] OR relax*[Other Term] OR meditat*[Other Term] OR stress*[Other Term]) AND (cancer[Title/Abstract] OR oncolo*[Title/Abstract] OR neoplasm*[Title/Abstract] OR tumor*[Title/Abstract] OR tumour*[Title/Abstract] OR Neoplasms[MeSH Terms] OR cancer[Other Term] OR oncolo*[Title/Abstract] OR neoplasm*[Other Term] OR tumor*[Other Term] OR tumour*[Other Term]) AND (randomized controlled trial[Publication Type] OR randomized[Title/Abstract] OR placebo[Title/Abstract] OR intervention[Title/Abstract] OR trial[Title/Abstract] OR “Clinical Trial” [Publication Type])
**Embase**
(diet:ab,ti,kw OR dietary:ab,ti,kw OR diets:ab,ti,kw OR nutrition:ab,ti,kw OR eating:ab,ti,kw OR 'nutrition'/exp OR 'diet therapy'/exp OR ‘physical activity’:ab,ti,kw OR ‘physical fitness’:ab,ti,kw OR exercis*:ab,ti,kw OR 'exercise'/exp OR ‘physical activity'/exp) AND (‘mind-body’:ab,ti,kw OR ‘mind body’:ab,ti,kw OR mindful*:ab,ti,kw OR relax*:ab,ti,kw OR meditat*:ab,ti,kw OR stress*:ab,ti,kw OR 'alternative medicine'/exp OR 'mental stress'/exp) AND (‘cancer’:ab,ti,kw OR oncolo*:ab,ti,kw OR ‘neoplasm*’:ab,ti,kw OR ‘tumor*’:ab,ti,kw OR ‘tumour*’:ab,ti,kw OR 'malignant neoplasm'/exp) AND (random:ab,ti OR placebo:ab,ti OR double-blind:ab,ti OR intervention:ab,ti OR trial:ab,ti OR ‘clinical trial’/exp OR ‘intervention study’/exp) AND 'article'/it
**PsycInfo**
TI diet OR AB diet OR TI dietary OR AB dietary OR TI diets OR AB diets OR TI nutrition OR AB nutrition OR TI eating OR AB eating OR DE “Diets” OR DE “Nutrition” OR DE “Eating Behavior” OR DE “Health Behavior” OR TI “physical activity” OR AB “physical activity” OR TI “physical fitness” OR AB “physical fitness” OR TI “exercis*” OR AB “exercis* OR DE “physical activity” OR DE “physical fitness” OR DE “Exercise”) AND (TI “mind-body” OR AB “mind-body” OR TI “mind body” OR AB “mind body” OR TI mindful* OR AB mindful* OR TI relax* OR AB relax* OR TI meditat* OR AB meditat* OR stress* OR AB stress* OR DE “Mindfulness” OR DE “Stress”) AND (TI cancer OR AB cancer OR TI oncolo* OR AB oncolo* OR TI neoplasm* OR AB neoplasm* OR TI tumor* OR AB tumor* OR TI tumour* OR AB tumour* OR DE Neoplasms) AND (TI random OR AB random OR TI placebo OR AB placebo OR TI double-blind OR AB double-blind OR TI intervention OR AB intervention OR TI trial OR AB trial OR DE “Randomized Clinical Trials”)
